# Characterization of 14-3-3 Proteins from *Cryptosporidium parvum*

**DOI:** 10.1371/journal.pone.0014827

**Published:** 2011-08-11

**Authors:** Stephen J. Brokx, Amy K. Wernimont, Aiping Dong, Gregory A. Wasney, Yu-Hui Lin, Jocelyne Lew, Masoud Vedadi, Wen Hwa Lee, Raymond Hui

**Affiliations:** 1 Structural Genomics Consortium, University of Toronto, Toronto, Ontario, Canada; 2 Structural Genomics Consortium, University of Oxford, Headington, Oxford, United Kingdom; Griffith University, Australia

## Abstract

The parasite *Cryptosporidium parvum* has three 14-3-3 proteins: Cp14ε, Cp14a and Cp14b, with only Cp14ε similar to human 14-3-3 proteins in sequence, peptide-binding properties and structure. Structurally, Cp14a features the classical 14-3-3 dimer but with a uniquely wide pocket and a disoriented RRY triad potentially incapable of binding phosphopeptides. The Cp14b protein deviates from the norm significantly: (i) In one subunit, the phosphorylated C-terminal tail is bound in the binding groove like a phosphopeptide. This supports our binding study indicating this protein was stabilized by a peptide mimicking its last six residues. (ii) The other subunit has eight helices instead of nine, with αA and αB forming a single helix and occluding the peptide-binding cleft. (iii) The protein forms a degenerate dimer with the two binding grooves divided and facing opposite directions. These features conspire to block and disrupt the bicameral substrate-binding pocket, suggesting a possible tripartite auto-regulation mechanism that has not been observed previously.

**Enhanced version:**

**This article can also be viewed as an enhanced version in which the text of the article is integrated with interactive 3D representations and animated transitions. Please note that a web plugin is required to access this enhanced functionality. Instructions for the installation and use of the web plugin are available in Text S1.**

## Introduction

*Cryptosporidium parvum* is the *Apicomplexa*n parasite responsible for cryptosporidiosis, a water-borne disease affecting both humans and animals especially predominantly in developing countries [1], [2]. Although occasional outbreaks occur in North America and Europe, there are hundreds of millions of new infections every year in African and Asian regions where contaminated water supplies are common. The lack of clean water can sequester some populations in chronic infection, which has been found to result in stunted development amongst children [3], [4]. Because of potentially rapid propagation via water systems, *Cryptosporidium parvum* is also classified as a bioterror agent. Furthermore, this parasite commonly infects farm animals.

Treatment of cryptosporidiosis is limited to use of nitaoxanize and the antibiotic paramomycin, both limited in effectiveness and founded in an unknown mechanism of action. Modest understanding of biochemical pathways in the parasite, along with socioeconomic considerations, hampers development of new drugs targeted at the disease. The first major step towards solving this problem was taken with the publication of the genome of *Cryptosporidium parvum* in 2004 [5], [6], [7], serving up a rich database (www.cryptodb.org) to facilitate identification and characterization of protein families and pathways shared with humans as well as those unique to the parasite.

One protein family common to humans, *C. parvum* and in fact all eukaryotes is the group of tyrosine 3-monooxygenase/tryptophan 5-monooxygenase activation proteins (YWHA), also known as 14-3-3 proteins [8]. These chaperones are identified by their trademark all alpha-helical, dimeric structures. Protozoan 14-3-3 proteins have been the subjects of limited research, with their role in the life cycle and pathogenesis of parasites only beginning to emerge [9]. For example, the single 14-3-3 protein in *Giardia duodenalis* has been found to be modified post-translationally *in vivo* and involved in a number of cellular processes [10]. The genome of *Toxoplasma gondii*, another *Apicomplexa*n parasite, encodes four putative 14-3-3 proteins, at least one of which may be membrane associated and an excreted/secreted antigen, suggesting its possible role as a vaccine candidate [11].

In comparison, there is a vast trove of data and knowledge on human 14-3-3 proteins. The structures of the entire family of seven isoforms, namely YWHAβ, ε, η, γ, τ, σ, and ζ, have been determined by X-ray crystallography, with and without peptides that mimic segments of substrate proteins [12], [13]. All 14-3-3 proteins are dimers, with each subunit comprised of nine α-helices framing a peptide-binding cleft. The dimer is shaped like the letter “W” from one angle, with an un-partitioned bicameral pocket where a substrate protein is held at two typically phosphorylated sites in the two component clefts [12], [13].

There is a minimum of 57% sequence identity amongst the members in the human family of 14-3-3 proteins. The alignment pattern can be broken into various highly conserved sequence motifs, including (i) a triad of Arg-Arg-Tyr in the peptide-binding cleft that attracts a phosphoserine or phosphothreonine, (ii) a region on the third helix featuring in particular arginine and leucine (including the first arginine in the triad), and (iii) an acidic C-terminus rich in aspartic and/or glutamic acids. The significant level of conservation results in a family of highly congruent structures: To wit, in the peptide-bound conformation, the backbone of 14-3-3ζ deviates from that of each of the other six human 14-3-3 proteins by a RMSD value of less than 2 Å [12].

Functionally, mammalian 14-3-3 proteins are known to regulate a large variety of cellular processes such as core metabolism, cell cycle, protein localization and trafficking and signal transduction [14]. They mediate these events through interaction with a large number of target proteins. For example, the human γ isoform has been found to interact with 170 potential binding partners [15], while the *S. cerevisiae* 14-3-3 proteins Bmh1 and/or Bmh2 have been shown to bind to 271 protein targets in a phosphorylation dependent manner [16]. Study of such interactions often entails experimental determination of peptide binding properties, using for example mode I (RSX(pS)XP), mode II (RXXX(pS)XP) and mode III peptides ((pS/pT)X_(1–2)_-COOH) [17] as well as the *Pseudomonas aeruginosa* Exoenxyme S toxin [18].

In addition to being helical dimers, all known 14-3-3 crystal structures share another characteristic: the C-terminus is missing either due to instability or strategic truncation (to optimize crystal formation). Consequently, the function of this terminus of 14-3-3 proteins remains the subject of conjecture, although a regulatory role has been implicated. Specifically, studies have shown that the truncation of this disordered C-terminus increases peptide-binding affinity of *Arabidopsis thaliana* YWHA*ω* [19] and human YWHAζ [20]. Furthermore, fluorescence resonance energy transfer (FRET) analysis has shown that C-terminus of YWHAζ is displaced when the protein binds a consensus peptide [21]. Although these are interesting experimental findings, direct structural evidence has thus far been unavailable.

To date, there is no published research on 14-3-3 chaperones from *Cryptosporidium parvum*. The genome of the parasite contains at least three genes encoding putative 14-3-3 proteins [5]. We have expressed all three proteins using a previously reported heterologous expression platform [22]. Herein, we present a study on their peptide-binding characteristics and molecular structures.

## Results and Discussion

### Identification of *Cryptosporidium parvum* 14-3-3 Proteins

The three *C. parvum* genes cgd3_1290, cgd7_2470 and cgd1_2980, encoding putative 14-3-3 proteins, were identified using a BLAST search [23] of the *C. parvum* genome [5] and the CryptoDB database - www.cryptodb.org [7]. There is also a 225-kDa protein (encoded by the gene cgd6_730) predicted to have a 14-3-3 domain, which is not included in this study.

**Figure 1:**
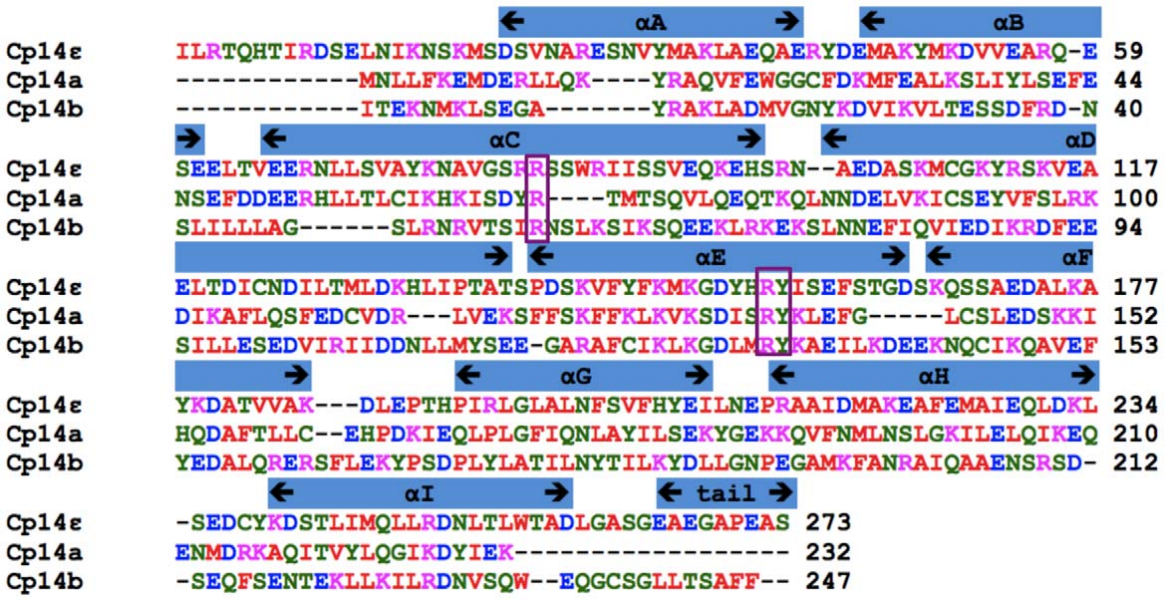
Sequence alignment. Sequence alignment of the three *C. parvum* 14-3-3 proteins. Sequence alignment shows positions of the helices. Alignment was performed using ClustalW. The key Arg-Arg-Tyr triad is highlighted by boxes. Of note is the absence of a C-terminus in Cp14a.

The closest human 14-3-3 homolog of the protein encoded by cgd3_1290 is the ε isoform, with 65% amino acid identity over 240 amino acid residues. Consequently, this parasitic protein is dubbed Cp14ε. The proteins encoded by the genes cgd1_2980 and cgd7_2470 are more distantly related to known 14-3-3's, both being less than 30% identical in sequence to the closest human homologues or any other protein outside the *Cryptosporidium* genus. Furthermore, the three *C. parvum* 14-3-3 proteins share no more than 27% sequence identity with each other. We have named the two most unique proteins Cp14a (cgd7_2470) and Cp14b (cgd1_2980). The alignment of the amino acid sequences of Cp14ε, Cp14a and Cp14b is shown in Fig. 1.

### Heterologous Expression and Peptide-binding Study

Using our heterologous expression platform [24], the three *C. parvum* 14-3-3 proteins yielded pure soluble protein of full length as well as constructs minimally truncated at either or both ends. All the samples eluted as stable dimers from gel filtration columns.

**Figure 2:**
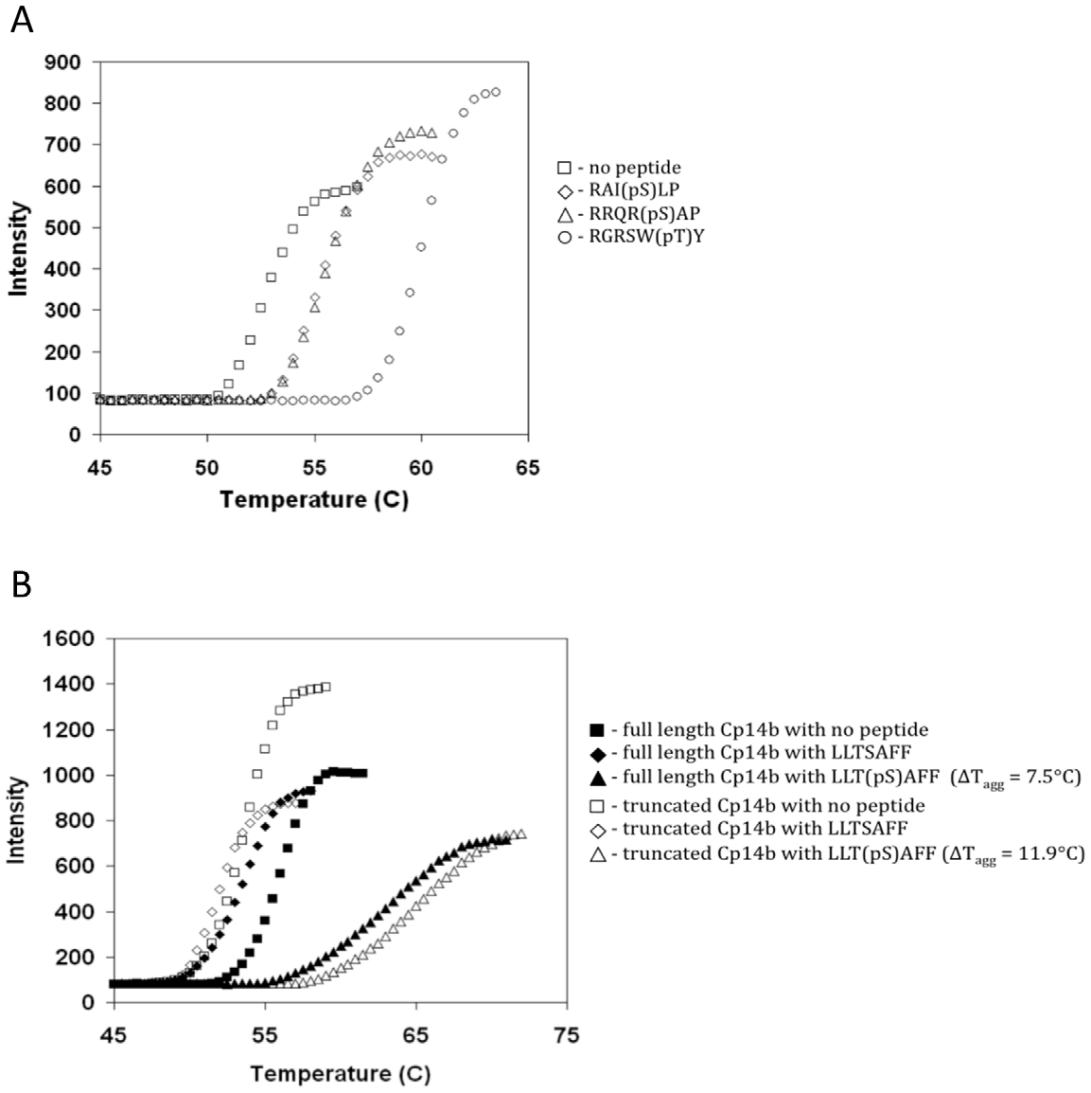
Peptide binding specificity of *C. parvum* 14-3-3 proteins. (A) Binding of Cp14ε to well-known consensus peptides: Thermostability of Cp14ε in the absence of any peptide (□) and in the presence of a 3-fold molar excess of RAI(pS)LP (◊), RRQR(pS)AP (Δ) or RGRSW(pT)Y (○) were assessed by DSLS. (B) Binding of Cp14b to peptides mimicking its own C-terminus: DSLS experiments were performed with full-length protein (closed symbols) and C-terminally truncated Ile1-Leu241 protein (open symbols) with no peptide added (▪ , □) and 2-fold molar excess of LLTSAFF peptide (♦, ◊) or LLT(pS)AFF peptide (▴,Δ). Thermostability experiments (DSLS) were performed using StarGazer as described in material and methods.

We applied the technique of differential static light scattering (DSLS) [25] to study the peptide binding characteristics of the *C. parvum* 14-3-3 proteins. This technique provides thermostability profiles of a number of proteins in parallel by measuring protein aggregation as a function of rising temperature. Unlike differential scanning calorimetry, DSLS does not provide quantitative thermodynamic data; however, it is a proven technique for high throughput identification and comparing the affinity of stabilizing ligands [24], [25]. In our experiments, a full-length protein sample of Cp14ε was initially found to be highly stable and absent of detectable aggregation up to 80°C. Subsequently, changing the buffer from pH 7.5 to 7.0 and using a shorter construct (Ile1-Asp259) yielded a less thermostable sample with a typical sigmoidal thermoaggregation profile suitable for ligand binding studies (Fig. 2A). Accordingly, a shorter construct was found to be stabilized by (and thus likely to bind) consensus peptides 1, 2 and 3 – i.e. RAI(pS)LP, RRQR(pS)AP, and RGRSW(pT)Y, respectively (Figure 2A). Using the same screening technique, all three peptides were found to have no effect on thermostability of Cp14a and Cp14b, indicating no interaction between these proteins and the common consensus peptides.

When screened against a peptide mimicking the last six amino acids at its C-terminus - LLSAFF, Cp14b was not stabilized by the non-phosphorylated sample but markedly stabilized by the phosphorylated peptide – LLpSAFF (Figure 2B). When the experiment was repeated with a sample of the protein with the last six amino acids truncated used instead of the full-length protein, there was a significant increase in the stabilization effect: an increase in T_agg_ of 11.9°C, compared to a 7.5°C increase for the full-length protein. This suggests that Cp14b not only binds the phosphorylated C-terminal mimetic but does so more readily without the interference of the actual terminal tail.

### Crystallographic Structures

Purified samples of all three *C. parvum* 14-3-3 proteins crystallized under conditions described in Table 1. The structural solution for Cp14ε was obtained from a 2.5 Å resolution dataset, using human 14-3-3σ (PDB accession 1YWT) as a model for molecular replacement, refined using common techniques, and submitted to the Protein Data Bank (PDB accession 2NPM). In the case of Cp14a, crystals of SeMet-labeled protein produced SAD data from which suitable phases were obtained; the resulting model was refined to 1.8 Å (PDB accession 2O8P). The 2.1Å structure for Cp14b (PDB accession 3EFZ) was obtained using a combination of SeMet phasing and a second, higher resolution dataset obtained from native crystals. All relevant crystallographic statistics are provided in Table 1.

**Table 1:** Crystallographic statistics of all three 14-3-3 structures: data collection, phasing, and refinement statistics.

		SeMet-labeled Cp14a	Native Cp14b	SeMet-labeled Cp14b	Cp14ε
**Crystallization Conditions**		15% PEG3350, 0.3 M ammonium acetate, and 0.2 M sodium citrate pH 5.6; 20°C	16% PEG3350, 10% ethylene glycol and 0.1 M HEPES pH 7.0; 4°C	14% PEG2000 monomethyl ether, 0.1 M TMANO, and 0.1 M Tris-HCl, pH 8.5; 20°C	14% PEG3350, 0.1 M calcium acetate, 0.2 M trimethylamine-*N*-oxide (TMANO), and 0.1 M HEPES pH 7.0; 20°C
Data Collection					
Space Group		I422	P21	P21	p41212
Cell Dimensions					
	a (A)	87.33	76.46	75.80	104.13
	b (A)	87.33	106.71	104.55	104.13
	c (A)	167.21	92.51	94.25	148.88
	alpha	90	90	90	90
	beta	90	112.83	114.81	90
	gamma	90	90	90	90
				Peak	
Wavelength		0.97918	1.54178	0.97942	1.0000
Resolution		50–1.82	50–2.08	50–2.9	50–2.52
Measured reflections		258621	156868	599374	407670
Unique reflections		28702	81493	29725	28423
Rsym		10.5(85.9)	11.9(87.2)	13.2(60.9)	6.3(98.1)
I/sigI		23.3(2.10)	12.1(1.21)	12.2(2.4)	10.4
Completeness (%)		98.1(87.1)	99.6(96.9)	99.8 (97.6)	99.9
Redundancy		9.0(6.3)	3.5(3.0)	7.1 (5.5)	14.3
Phasing					
FOM SOLVE		0.365		0.176	
FOM RESOLVE		0.766		0.698	
FOM RESOLVE_BUILD				0.912	
Refinement					
Resolution		50–1.82	25.0–2.08		42.7–2.52
Number of Reflections		27920	77311		27756
Test Set		757	4081		599
Rwork/Rfree		19.8/23.9	22.5/26.7		22.1/27.5
Number of Atoms					
Protein		1848	7172		3611
Water		235	420		110
Ligand			36		40(peptide)
Mean Bfactor		22.91	37.6		28.69
Ramachandran Favored		96.4	97.75		94.2
Ramachandran Disallowed		0	0		0
RMS deviations					
	Bond lengths (A)	0.013	0.011		0.016
	Bond Angles	1.204	1.246		1.498

**Figure 3:**
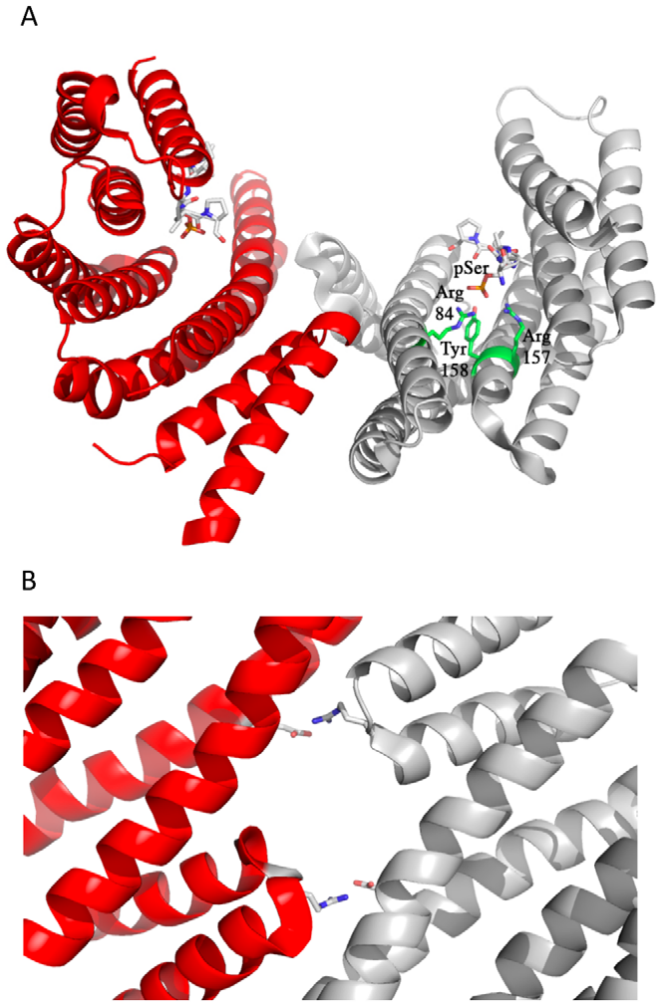
Cp14ε dimer. (A) This figure depicts the structure of Cp14ε which is a conventional 14-3-3 dimer in every respect, from structure to peptide-binding to dimerization interface. One subunit is in red while the other is in gray. The peptide RAI(pS)LP is bound in each binding groove. The Arg-Arg-Tyr triad is shown in the gray subunit. This and subsequent figures of protein structures were rendered using the program PyMol (Delano Scientific, Palo Alto, CA, USA). (B) Dimerization interface between the two subunits of Cp14ε features the same Arg-Glu salt bridges previously identified in human 14-3-3 proteins.

With Cp14ε being close to or over 60% identical to most human 14-3-3 proteins, its structure is expectedly congruent to the human homologues (see Fig. 3A), deviating from the 14-3-3ε (2BR9), η (2C74), σ (1YWT), γ (2B05), ζ (1QJA), τ/θ (2BTP) and β (2C23), structures by root mean square (RMS) values of 0.9, 1.1, 1.2, 1.2, 1.5, 1.6 and 1.9 Å respectively, as measured in the program Pymol (Delano Scientific, CA). With such a high degree of similarity, it is not surprising that the parasitic chaperone is stabilized by and therefore predicted to bind mode I, II and III peptides (Fig. 2A), just like the human homologue. It was crystallized with the mode I peptide RAI(pS)LP bound. The triad Arg84, Arg157 and Tyr158 (numbering based on model with PDB accession 2NPM) are in their typical positions and orientations to coordinate the binding of the phosphoryl moiety of the peptide (Figure 3A), as is the case in all other 14-3-3 structures with phosphopeptide bound [13], [17]. Other common 14-3-3 structural motifs are also found here. On the third helix, the sequence RNLLSVAYKNAVGSRR (ending in Arg84) is largely similar to that found on the human homologues, familiarly featuring multiple arginines and leucines, as well as a valine near the end. Following helix α9, there is also an acidic C-terminus featuring glutamic acid - EAEGAPEAS, although it is truncated in the crystallized construct in a successful attempt to optimize crystal diffraction and therefore absent in the structure. The mode of dimerization is also well conserved, with an aperture formed at the subunit interface. The key salt bridge between the two protomers, previously identified in the human structures [12], [13], is conserved in Cp14ε as Arg43-Glu119 (Figure 3B). Overall, this is a typical 14-3-3 protein sharing most of the common sequence and structural motifs seen in previously studied homologs.

**Figure 4:**
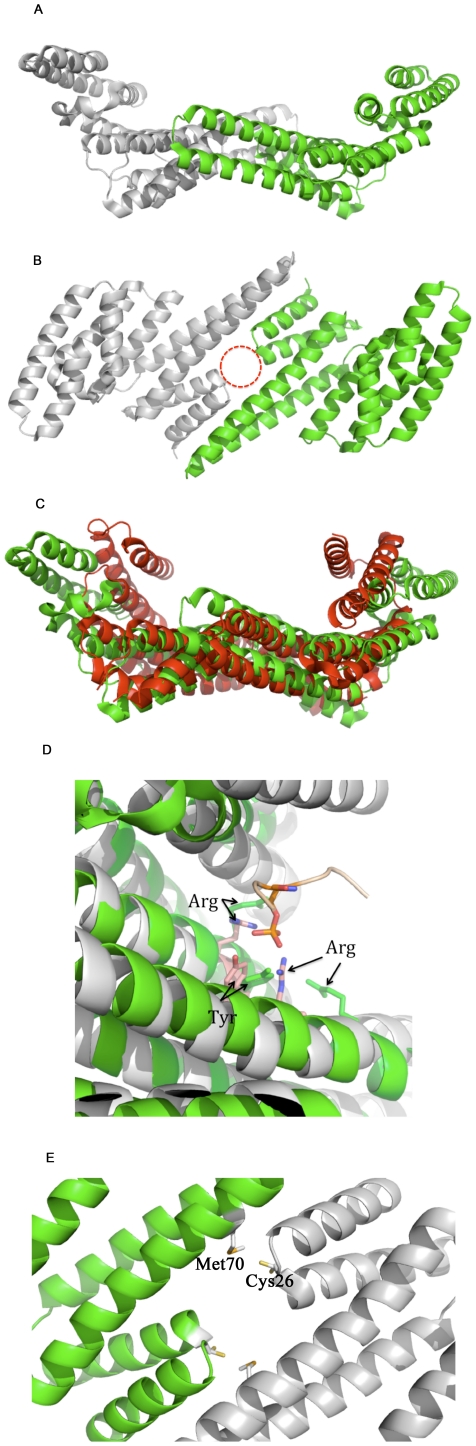
Cp14a dimer. (A) Structure of Cp14a dimer shows a uniquely wide substrate-binding cradle. (B) Alternate view of Cp14a shows the familiar aperture (highlighted as a red circle) at the “pit” of the bicameral substrate-binding pocket. The absence of a C-terminal tail is confirmed in both Figs. 4A and 4B, even though the protein is fully crystallized and stable in this region. (C) Superposition of Cp14a (green) on the more typical Cp14ε (red) shows that the former is notably wider than the latter. (D) The Arg-Arg-Tyr triads of Cp14a (green) and Cp14ε (pink) superimposed together show that the three residues from Cp14a are farther apart and oriented away from typical phosphoserine binding site. The phosphorylated ligand shown is from the Cp14ε structure. Since Cp14a was not stabilized by any of the three ligands used in a DSLS-based thermostability based assay, this brings into question whether this protein binds phosphopeptides, if at all, in a different way than Cp14ε and other previously studied 14-3-3 proteins. (E) The Cp14a dimer is not held together by the Arg-Glu salt bridges seen in other 14-3-3 proteins (Fig. 3B). Instead, they are replaced by Cys-Met hydrophobic pairs.

As mentioned above, Cp14a shares limited sequence identity with Cp14ε and all other known proteins. This divergence is manifested in our crystallographic structure of this protein in a number of ways (Figure 4; PDB ID 2O8P). First, while the Cp14a structure also adopts the classic dimeric α-helical structure, it stands out by virtue of its unusual width (Figs. 4A, 4B and 4C). On the third helix, RHLLTLCIKHKISDYR (ending in Arg68 of the triad; numbering based on the model with PDB accession 2O8P) is the sequence, which retains the commonly found arginines and leucines but deviates in other positions, missing in particular the aforementioned valine. More strikingly, Cp14a is further distinguished by the natural absence of a C-terminal tail ensuing the last helix (Figs 4A, 4B and Fig. 1). While the putative phosphopeptide binding triad is apparently conserved as Arg68, Arg136, and Tyr137 (Figure 4D; amino acid numbering based on the PDB model 2O8P), Arg68 appears distant and points away from the other two residues. Even the second arginine and the ensuing tyrosine are skewed in their orientations with respect to what is typical in other 14-3-3 proteins, with or without ligands bound. This is the first known instance of a disoriented 14-3-3 triad (superposed on Cp14ε for comparison in Fig. 4D). Together with Cp14a's unusually wide binding pocket and our binding study suggesting its lack of affinity for the three mode I, II and III peptides tested, this brings into question about whether and how this protein might interact with phosphopeptides.

The mode of dimerization of Cp14a deviates from that of other 14-3-3 proteins. While the two subunits form an aperture similar to that in the human 14-3-3 proteins and Cp14ε (Fig. 4B), the side chain interactions around the aperture are primarily hydrophobic, with Cys26 and Met70 of opposite chains occupying the position of the Arg-Glu salt bridge conserved in most other homologs (Fig. 4E). Overall, Cp14a is a novel and unique 14-3-3 protein, with its crystallographic structure providing more questions than answers about its binding properties and function.

While Cp14ε conformably binds a number of known 14-3-3 ligands and Cp14a attracts none, the third *C. parvum* 14-3-3 protein in our study stands out by interacting with a peptide that is a phosphorylated copy of the last six amino acids in its own C-terminus: LLT(pS)AFF – a motif hitherto not known to be relevant in the context of 14-3-3 proteins. Specifically, as shown in our thermostability study (Fig. 2B), Cp14b was most strongly stabilized by a form of this peptide with the serine residue phosphorylated when its own tail was truncated, suggesting competition between the C-terminus and its septuplet mimic when both are present. This interaction is corroborated and elucidated by our crystallographic structure of the enzyme.

**Figure 5:**
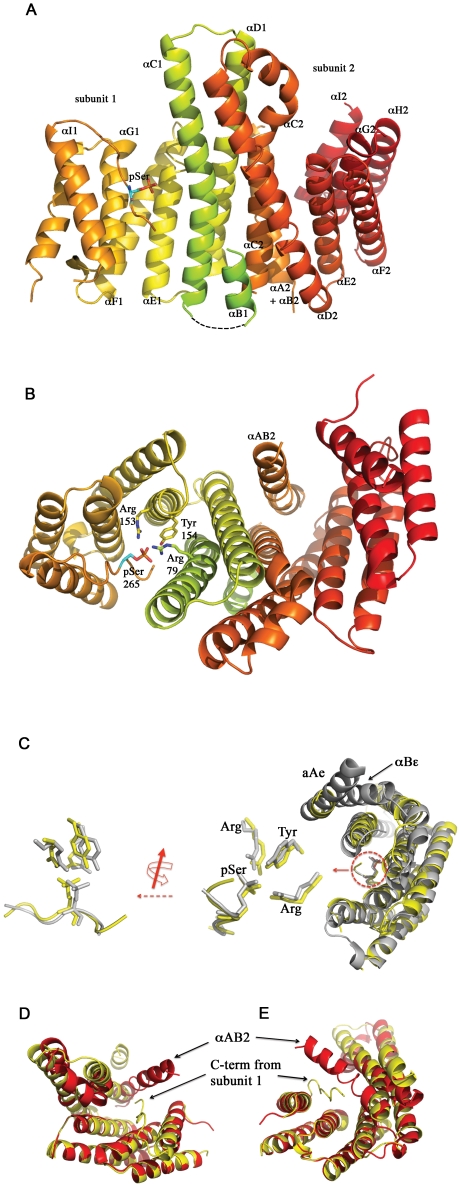
Structure of Cp14b. (A) Overall structure of Cp14b shows two subunits. Subunit 1 has 9 helices just like other 14-3-3 proteins. The first helix A1 is partly disordered and only visible in the model as a relatively short segment. Furthermore, it can be seen that the C-terminus of this subunit is bound in its peptide-binding groove, with a phosphoserine clearly showing. The second helix αB1 is completely disordered and therefore not visible. Subunit 2 only has 8 helices, as αA2 and αB2 are a single continuous superhelix. There is nothing bound in the peptide-binding groove in this protomer. (B) Alternate view of Cp14b with protomer 1 shows that its tail is in its peptide-binding groove and interacts with the Arg-Arg-Tyr triad in the typical 14-3-3 fashion. The superhelix αAB2 in protomer 2 is more clearly visible in this view. (C) The peptide-binding groove in subunit 1 of Cp14b (yellow) is superimposed on that of Cp14ε (gray), illustrating that Cp14b interacts with its tail in the same way as Cp14 does with a phosphopeptide. Specifically, the ligand sites are magnified and rotated to show two views: In one view (middle), the phosphoserine in the Cp14b tail (yellow) and in the phosphopeptide in the Cp14ε complex (gray) are aligned, as are the Arg-Arg-Tyr triads. In the left magnified view, we can see that the tail of Cp14b (yellow) is roughly aligned with the backbone of the phosphopeptide in the Cp14ε complex. Furthermore, it can be seen that the first helix (αA1 from Cp14a and αAε from Cp14ε) of the two *C. parvum* 14-3-3 proteins are roughly aligned, while αB1 from Cp14b is disordered and therefore not visible. (D) and (E) Two views of one subunit of Cp14b superimposed on the other to show their differences in the peptide-binding cleft: In one subunit (red), the superhelix AB2 blocks the peptide-binding site, which is open in subunit 1 (yellow) so as to permit interaction with its own C-terminus.

As shown in Figs. 5A and 5B, Cp14b is a dimer of two bundles of anti-parallel helices just like all other 14-3-3 proteins. Beyond this, we can see a number of significant departures. First, the two protomers, numbered 1 and 2 with their helices accordingly labeled in Fig. 5A, are not identically folded (see Figs. 4D and 4E for superposition of the subunits on each other). Protomer 1 follows the standard 14-3-3 architecture, albeit with the helix αB1 atypically disordered (and hence not visible in the crystallographic structure). This protein is in a ligand-binding conformation but the ligand, instead of an extraneous peptide, is the protein's own C-terminus. The bound tail features a phosphorylated serine and, as noted above, the sequence LLT(pS)AFF. This serine, which was found to be phosphorylated in the crystal structure despite the fact that the protein was expressed in E. coli, interacts with the Arg79-Arg153-Arg154 triad just as the mode 1 peptide does in Cp14ε (Figs. 5B, 5C), with the backbone of Cp14b's tail tightly aligned with the peptide RAI(pS)LP (Fig. 5C) and fitting in the binding groove in a very similar way.

In contrast, the other subunit of Cp14b, namely protomer 2, has a disordered C-terminus in our crystallographic structure; however, this polypeptide displays its own unique feature. Specifically, the helices αA2 and αB2 appear as one continuous helix, behaving like a bent spring given room to relax and straighten (Figs. 5A and 5B). The resulting “super” helix, dubbed αAB2, is shifted from where αA and αB are typically found. In its new position, αAB2 blocks access to the binding groove and potentially contributes to inhibition of Cp14b's ability to bind another protein, as seen in Figs. 5D and 5E from two different angles.

With protomer 2 adopting a conformation with only 8 helices, Cp14b can no longer form a dimer in the same way as other 14-3-3 proteins, which rely on helices A and B configured as a bent spring. The novel dimer, held together by hydrophobic interaction, does not form a merged and undivided substrate cradle. Instead, the two binding grooves face opposite directions and are separated by helices αC1, αD1, αAB2, αC2 and αD2. The division and twisting of this cavity makes it impossible for the chaperone to hold a substrate in the standard way and potentially forms a third mode of inhibition for Cp14b.

### Diversity and Uniqueness of *C. parvum* 14-3-3 Proeins

Of the three *C. parvum* 14-3-3 proteins identified and discussed herein, only Cp14ε shares a significant level of sequence homology with 14-3-3 proteins from other species. In contrast, all 13 *Arabidopsis thanalia* 14-3-3 proteins are highly similar to human homologues[26]. In the genome of *Plasmodium falciparum*, which is found in the same *Apicomplexa* phylum as *Cryptosporidium*, there are two 14-3-3 proteins annotated. One of them is encoded by the gene MAL8P1.69, which is most similar in sequence to YWHAε from humans and *Arabidopsis* (data not shown). The second one is encoded by MAL13P1.209 and shares lower than 30% sequence identity with proteins outside the *Plasmodium* genomes, including *Cryptosporidium*.

Given its similarity to human and plant homologues, Cp14ε unsurprisingly binds all three consensus peptides. It is also structurally similar not only to human YWHAε (PDB ID 2BR9), but also to the 14-3-3 protein from the fungus *Nicotiana tabacum* (PDB ID 1O9C), with alignment deviation of 0.59 Å and 0.56 Å respectively.

In contrast, Cp14a is distinctive both in sequence comparison and in structure. Specifically, the exceptional width of its substrate pocket is unseen in all other available 14-3-3 structures with and without peptides available. One intuitive conclusion is that this protein supports a large substrate; however, its disoriented Arg-Arg-Tyr triad, the lack of a C-terminal tail and our negative screening results make this *C. parvum* chaperone even more divergent from others and introduces the possibility that this is a degenerate 14-3-3 protein, or at least one that is functionally unique.

As discussed above, Cp14b is unique in various ways. If a standard 14-3-3 dimer can be described as a pair of open arms flexed to embrace substrate proteins, then Cp14b has both arms blocked at the elbows and additionally twisted with respect to each other. Not only does this disrupt the bicameral substrate-binding pocket, each protomer is prevented from peptide-binding. Although the 14-3-3 C-terminus has been predicted to play a regulatory role [19], [20], [21], such a tripartite auto-inhibitory mechanism has not been presaged by any previous experimental results and can only be explained in the biological context of the parasite with further research. We can predict, however, most other 14-3-3 proteins are unlikely to bind their C-terminus in the same way as Cp14b. This is because the Cp14b does not contain mostly aspartic and glutamic acids in this region. Furthermore, our study showed that this parasitic chaperone only sequestered its own tail in the presence of phosphoserine. Most other 14-3-3 proteins do not contain a serine or threonine in this region. In conclusion, Cp14b's binding of its own C-terminus is a unique rather than common regulatory mechanism.

Helices αA and αB are essential to the formation of the standard 14-3-3 dimer with the cup-shaped substrate cradle, specifically the signature aperture at the bottom. In Cp14b, the division and distortion of the substrate cradle is precipitated by the “relaxation” of helices αA2 and αB2 into a single super helix. The possibility exists that 14-3-3 monomers naturally fold into eight helices, with the N-terminal helix bent sharply into two shorter, anti-parallel helices only during the formation of the dimer.

Research on 14-3-3 proteins is largely a study in conformity. Interestingly, it is from the compact genome of *C. parvum* that significant structural diversity is discovered. As mentioned above, there is also a *Plasmodium* 14-3-3 with a distinctive sequence. Further research is required to understand the biological implications of this divergence which has not been observed outside of Apicomplexan parasites.

## Methods

A thorough description of our protein expression and crystallography methods for parasitic proteins has been published [22]. In addition, protocols for preparing proteins leading to solved structures also available at the SGC website (www.thesgc.org).

### Materials

Peptides were purchased from the Advanced Protein Technology Centre, Hospital for Sick Children (Toronto, Ontario, Canada), or the Tufts University Core Facility (Boston, MA, USA). Genomic DNA for *Cryptosporidium parvum* strain Iowa was purchased from ATCC (Manassas, VA, USA). The *E. coli* strain BL21 (DE3)-R3 (with R3 denoting a mutant developed to resist a T1-like bacteriophage), generously donated by the Structural Genomic Consortium at Oxford University (Oxford, UK) and augmented by the plasmid pRARE2 isolated from the Rosetta2 cells from Novagen, was used for heterologous expression of the *C. parvum* 14-3-3 proteins in this study. All other materials, unless specified, were of the reagent grade purity or better.

### Cloning

The cgd1_2980, cgd3_1290, and cgd7_2470 genes (gene ID from www.cryptodb.org) were amplified from *Cryptosporidium parvum* strain Iowa genomic DNA by PCR using Platinum Pfx DNA polymerase (Invitrogen, Carlsbad, CA, USA) and cloned into the pET15 based vector p15TV-L by the ligation independent cloning method. To increase probability of successful expression and crystallization, multiple constructs per target protein were designed using the PsiPred software [27] to predict each protein's secondary structure. Each protein clone had an N-terminal addition of MGSSHHHHHHSSGRENLYFQ*G to include a hexahistidine tag and a tobacco etch virus (TEV) protease cleavage site indicated by an asterisk.

### Protein expression and purification

The cloned constructs were transformed into BL21 (DE3)-R3-pRARE2 cells. The transformed cells were grown in a Lex-48 high-throughput bioreactor system (Harbinger Biotechnology and Engineering Corp., Markham, ON, Canada). The Cp14ε protein was grown in TB media, while Cp14a and Cp14b were grown in 2×2 L selenomethionine-containing M9 minimal medium (kit from Medicilon, Inc, Chicago, IL, USA). Cultures were initially grown at 37°C until late-log phase (typically around OD 5) and cooled to 15°C. Subsequently, 0.4 mM IPTG was added to induce expression and the cultures were left to grow for 16 h. Cell pellets were collected by centrifugation, re-suspended, flash frozen in liquid nitrogen and stored at −80°C.

Prior to protein purification, cell pellets were thawed overnight at 4°C. The cells were treated with 0.5% (w/v) 3-[(3-Cholamidopropyl) dimethylammonio]-1-propanesulfonate (CHAPS) and 2000 U benzonase, and then mechanically lysed using a Microfluidizer M-110EH processor (Microfluidics Inc.) at 18,000 psi. Lysates were centrifuged at 70,000× g for 20 min. Cleared lysates were applied to 20 mL DE-52 anion exchange resin (Whatman) followed in series by 2 mL Ni-NTA Superflow resin (Qiagen). The Ni-NTA resin was washed with 200 mL buffer A containing 30 mM imidazole, and protein was then eluted with 15 mL buffer A containing 250 mM imidazole. Protein samples were treated with 1 mM EDTA and 2 mM DTT, then applied to a Superdex-S200 26/60 column (GE Healthcare) and gel filtration runs were carried out using *crystal buffer* (10 mM HEPES pH 7.5, 500 mM NaCl).

For Cp14ε and Cp14a, the best crystals were obtained with the N-terminal histidine tag removed. To achieve this, in-house purified histidine tagged TEV protease was added in a pre-determined molar ratio. The solution was incubated at 4°C overnight, and then the untagged protein was separated from the cleaved tag and TEV protease by passage through 1–2 mL of Ni-NTA resin.

### Peptide binding analysis

Purified proteins were screened against potential binding peptides by thermostability analysis using the StarGazer™ instrument (Harbinger Biotechnology and Engineering Corp.), which compares the stabilizing effects of different ligands on proteins based on differential static light scattering (DSLS) [24] [24], [25]. The *C. parvum* 14-3-3 proteins were screened using this technique against the consensus peptides (I, II and III) at different concentration ratios. The Cp14b protein was also screened against the peptides LLTSAFF and LLT(pS)AFF, which mimic the last 7 amino acids at its own C-terminus. Relative binding affinity was analyzed by comparing the thermoaggregation curves in the presence and absence of peptides. The point of inflection of each curve was identified as *T*_agg_ (aggregation temperature). Increase in *T*_agg_ – i.e. ΔT_agg_ – in the presence of a ligand is used a measure of the stabilizing effect of the ligand [24], [25].

### Protein crystallization

Cp14ε was first incubated on ice for 1 h with consensus peptide I RAI(pS)LP in a 3∶1 (peptide:protein) molar ratio. The Cp14ε:peptide complex was crystallized using a reservoir buffer of 14% PEG3350, 0.1 M calcium acetate, 0.2 M trimethylamine-*N*-oxide (TMANO), and 0.1 M HEPES pH 7.0, with 1 µL protein solution (8 mg/mL) added to 1 µL reservoir buffer in a hanging drop at 293 K. Diamond shaped crystals (∼200 µm) were soaked for ∼1–2 min in a cryo solution containing reservoir buffer diluted by the addition of glycerol to 20% (v/v), and then flash-frozen in liquid nitrogen.

A truncated construct of SeMet-labeled Cp14a (E6-K238) was crystallized using the hanging drop at 277 K with 1 µL of 8 mg/mL protein mixed with 1 µL reservoir buffer containing 15% PEG3350, 0.3 M ammonium acetate, and 0.2 M sodium citrate pH 5.6. Small (100 µm) cubic crystals were soaked for ∼1–2 min in a cryo solution containing reservoir buffer diluted by the addition of glycerol to 30% (v/v), followed by flash-freezing in liquid nitrogen.

For the purpose of removing partially aggregated protein, a sample of native Cp14b was first added to a solution of 14% PEG3350, 10% ethylene glycol and 0.1 M HEPES, pH 7.0 in a ratio of 3∶2 protein:buffer, and incubated at ∼20°C for 15 min. The mixture was then centrifuged at 18,000 x g for 15 min, with the supernatant applied to a coverslip in 3 µL hanging drops and placed over reservoir buffer containing 16% PEG3350, 10% ethylene glycol and 0.1 M HEPES pH 7.0, and incubated at 20°C. Large (500 µm) crystal plates were first dehydrated by exposure of the drop, untouched, to air for ∼15 min before harvesting of the crystal and mounting directly on a nitrogen cryo stream. The selenomethionine substituted Cp14b protein was crystallized at 293 K with 1 µL protein (8 mg/mL) and 1 µL reservoir buffer (14% PEG2000 monomethyl ether, 0.1 M TMANO, and 0.1 M Tris-HCl, pH 8.5). Plate crystals were soaked in a cryo solution consisting of reservoir diluted by addition of ethylene glycol to 20% (v/v) and flash frozen in liquid nitrogen.

### Crystallography data collection and structure determination

For Cp14ε and Cp14a, native and SeSAD data were respectively collected at the Advanced Photon Source, beamline 17-ID with an ADSC QUANTUM 4 CCD detector, and processed and scaled with HKL2000 [28]. A molecular replacement solution for Cp14ε was found using the human sigma isoform of 14-3-3 structure, PDB code 1YWT [29] as a starting model and the program PHASER [30]. Model building and refinement were carried out in COOT 0.1.2 [31] and REFMAC 5.2 [32] programs, respectively.

For Cp14a, the program suite SOLVE/RESOLVE was used to solve the SAD phases at the Se peak. Model building and refinement were carried out in COOT 0.1.2 [31] and REFMAC 5.2 [32] programs, respectively. A SeSAD data set was collected for Cp14b at beamline A1 of the Cornell High Energy Synchrotron, with an ADSC QUANTUM 210 detector. Data were collected over 360 degrees at 1 degree oscillations, then indexed and scaled using the program HKL2000 [28]. The program SOLVE [33], [34] was used to find a single selenium site and create phases to 3.4 Å resolution. The resulting phases were poor, but after density modification using RESOLVE [33], [34] and non-crystallographic symmetry averaging, the resulting map revealed discernable helical structure. A higher resolution native data set was collected on a dehydrated crystal using home source Rigaku rotating anode FRE and plate detector. Data were indexed and scaled with HKL2000 [28]. The initial phases were combined with native data and RESOLVE_BUILD [33], [34] was used to update and improve an initial model of helices. All model building and refinement were done using the COOT graphics interface and the CCP4 suite of programs.

## Supporting Information

Text S1 Instructions for installation and use of the required web plugin (to access the online enhanced version of this article).
(PDF)

Datapack S1 Standalone iSee datapack - contains the enhanced version of this article for use offline. This file can be opened using free software available for download at http://www.molsoft.com/icm_browser.html.
(ICB)
